# Epigenetic Regulation of Development, Cellular Differentiation, and Disease Progression/Protection in Adults

**DOI:** 10.3390/cells11121907

**Published:** 2022-06-12

**Authors:** Rebecca J. Ryznar, Lacie Phibbs, Erin Onat, Lon J. Van Winkle

**Affiliations:** 1 Department of Biomedical Sciences, Rocky Vista University, Parker, CO 80134, USA; 2 College of Osteopathic Medicine, Rocky Vista University, Parker, CO 80134, USA; lacie.phibbs@rvu.edu (L.P.); erin.onat@rvu.edu (E.O.); 3 Department of Medical Humanities, Rocky Vista University, 8401 S. Chambers Road, Parker, CO 80112, USA; lvanwi@midwestern.edu; 4 Department of Biochemistry, Midwestern University, Downers Grove, IL 60515, USA

Epigenetic changes drive early embryonic and later stages of development. Through this molecular mechanism, transcriptional programs are altered, influencing outcomes for cellular differentiation patterns in early life and throughout adulthood [1]. A multitude of epigenetic modifications are necessary for successful development throughout early life and are associated with a youthful, healthy epigenetic landscape, but age-related and environmentally induced epigenetic changes can cause a multitude of pathologies in adults [1,2,3,4]. As such, an epigenetic clock can reflect changes that occur with aging and environmental stressors [5]. As we age, there is evidence of a general loss of histones, transcriptional amplification, changes in heterochromatic regions, and methylation patterns [2]. The epigenetic clock has been reported to capture aspects of biological aging and its associated morbidity and mortality and can even be used to predict age [6]. Trauma and chronic stress have also been linked to changes in our epigenetic clock [7].

Alzheimer’s disease, cancer, cardiovascular disease, and diabetes are some of the more well-studied diseases associated with an aged epigenetic landscape [8,9,10,11,12,13]. Studies have even linked prognosis following cancer diagnosis with the extent of an aged epigenetic clock [14]. Similarly, autism spectrum disorder may be associated with a brain epigenome that is gender-specific [15], while epigenetics involving miRNAs help to cause obesity due to early life stress [16]. Such long-term health risks are also associated with epigenetic changes due to maternal diet and assisted reproductive technology [17]. Epigenetic changes even occur in transposable elements [18], and these, as well as other epigenetic modifications, may alter one’s personality [19] and initiate neuropsychiatric disorders [20].

This Special Issue aims to explore current research concerning epigenetic changes that govern human development, both embryonic and later cell stages, along with age-related epigenetic changes that drive pathologies later in life. We invite the submission of manuscripts that concern epigenetic contributions to development, aging, and transgenerational inheritance. Additional manuscript topics include, but are not limited to, embryonic development, differentiation, metabolic signaling, DNA methylation, histone modifications, miRNAs, transposable elements, and the epigenetic clock. Finally, manuscripts regarding possible treatment targets and early intervention via the modification of these molecular mechanisms are also welcomed, e.g., [21].
